# Expansion and evolution of the R programming language

**DOI:** 10.1098/rsos.221550

**Published:** 2023-04-12

**Authors:** Timothy L. Staples

**Affiliations:** School of Biological Sciences, The University of Queensland, Building 60, St Lucia, Queensland 4072, Australia

**Keywords:** software linguistics, programming functions, lexicon, GitHub repositories

## Abstract

Languages change over time, driven by creation of new words and cultural pressure to optimize communication. Programming languages resemble written language but communicate primarily with computer hardware rather than a human audience. I tested whether there were detectable changes over time in use of R, a mature, open-source programming language used for scientific computing. Across 393 142 GitHub repositories published between 2014 and 2021, I extracted 143 409 288 R functions, programming ‘verbs’, pairing linguistic and ecological analyses to detect change to diversity and composition of functions used over time. I found the number of R functions in use increased and underwent substantial change, driven primarily by the popularity of the ‘tidyverse' collection of community-written extensions. I provide evidence that users can change the nature of programming languages, with patterns that match known processes from natural languages and genetic evolution. In R, there appear to be selective pressures for increased analytic complexity and R functions in decline that are not yet extinct (extinction debts). R's evolution towards the tidyverse may also represent the start of a division into two distinct dialects, which may impact the readability and continuity of analytic and scientific inquiries codified in R, as well as the language's future.

## 1. Background

The reality of a language is defined by the words that are used, not all the words that exist [1,2]. Languages change over time as new words are created and existing words die [3], driven by interacting social and cultural forces [1,4,5]. Words are born alongside new domains and knowledge, which require more specific and specialized terminology [6,7]. This increase in language size is counterbalanced by cultural pressure to use words that communicate effectively and efficiently [8]. Words with similar meanings compete [3]. While random drift in word use also occurs [9], generally, speakers align and converge their language use [10]. The most optimal word becomes dominant and redundant words go extinct [11,12], resulting in a reduction in language size [6,13]. Over time these processes cause languages to evolve and change [14], reflecting mechanisms of genetic evolution [2,5].

It is unclear whether these evolutionary patterns and mechanisms extend to designed languages, such as programming languages. Software linguistics, the application of linguistic tools and theories to software languages [15], has rarely been studied in earnest, and only with small datasets [16]. New programming languages are designed, become popular, and are eventually superseded; most research on software linguistics has focussed on this inter-language scale (e.g. [17]). Change in use may also occur within a programming language, but this hypothesis remains largely untested [16].

In some ways, software programs resemble books, which codify language to communicate to an audience [18–21]. Books communicate to people; programs communicate to computer hardware. The words and grammatical rules in natural languages proxy similar structures in programming languages [22]. Programming languages have strict grammatical rulesets, filled with objects like variables (nouns) and functions (verbs). But many of the processes that dictate evolution in natural languages do not have direct programming parallels. Computers have perfect comprehension of correctly specified programs, which may limit the cultural feedback that drives language convergence. So long as the use cases of software remain constant, we may expect the human-computer dynamic of programming languages to be resistant to change. However, software is still written by and read by humans. Intra-language change can be driven by top-down authorities [10], which may resemble formal updates to programming vocabulary and published style guides. Programming language change can also be unregulated and spontaneous [10], potentially driven by user-created extensions and cultural consensuses.

The popularity of version control hosting sites such as GitHub provides access to immense programming language corpora, making it possible to study broad patterns of change over time. Here, I pair linguistic and ecological perspectives to capture change in the diversity and composition of programming functions used in the R language. R is an ideal software language to test for evidence of language change. It is ranked in the top 20 most popular programming languages [23] and is free and open source, creating a broad user base. It is specifically targeted to data analysis and statistical inference, restricting language use cases [24]. R has been available since 1993, without separation of major versions, creating a continuous legacy of training and use in the scientific and data analytic fields. It also has a history of community-created ‘packages' of R software that extend and replace core functionality, which have been extremely popular with R users. This continued development cycle and user-led package development creates theoretical potential for the use of R to change and evolve over time.

Using 393 142 online repositories of R programs publicly housed in GitHub, treating R functions as words, I asked the following questions:

1. Has the diversity and composition of R functions in use changed over time, both across and within GitHub repositories?
2. To what extent has function use in R been supplemented by community-led development of packages?
3. Are there trends in function and package use that highlight how R is changing?

## 2. Methods

### 2.1. GitHub web scraping and data processing

#### 2.1.1. Data acquisition

I accessed repositories (repos) published on GitHub between 1st January 2014 and 31st December 2021 using the API. I identified all repos published each day (up to a maximum of 1000) with the R language flag. I then extracted every ‘.R' script file in each repo, a total of 5 443 762 scripts housed in 701 956 repos, authored by 243 208 different GitHub users. Within each script, I used a custom function-extracting algorithm that captured text to the left of an open parenthesis (‘(’), the syntax for a function call in R. In addition, I recorded instances of three common operators which act as functions with different syntax (%in%, [] and % > %). In total, these R functions are equivalent to 1-gram verbs commonly used in corpus linguistics studies [24]. This resulted in a dataset of 1 938 744 unique functions used a total of 254 738 400 times across all repos.

Next, I associated each function with its associated R package. This included the ‘base' set of packages that is automatically loaded alongside R, as well as many popular community packages that must be downloaded separately. To do this, I matched function names in the dataset with the list of functions in each package. This process operated without error for any function name that was unique across all packages. Some function names (e.g. ‘plot', ‘predict') were repeated across different packages (with different versions for specific object classes or contexts). The actual package used in these cases was obscured, so I attempted to identify the most likely package source. Where the function shared a name with a base function, I associated the function with the relevant base package, assuming that the function was most likely an extension of the base function applied in a package-specific context. This process was then repeated for all functions that did not match a base function, first for the set of ‘recommended' R packages. For community packages, I conducted an iterative process, whereby I identified the most common functions not associated with a base package, added the most likely package including that function, updated the list and repeated for the next most common function with no associated package.

#### 2.1.2. Data processing

I processed these raw data to better capture overarching trends in R function usage over time. First, I removed scripts from 138 872 repositories that were updated 12 months or more after their initial creation date. As I accessed R scripts in their most recent GitHub version but used the original repository creation date as a timestamp, these repositories were likely a poor representation of function use trends at the time the repository was created.

Second, I removed functions that were not associated with an R package: this was almost all functions (1 279 873: 98.80% of the total unique functions). These non-package functions came from several sources. First, I did not include functions from rarely used packages. Second, R functions are objects and can be created locally within scripts or sourced from other scripts *ad hoc*, without needing to be included in a published package. Finally, some functions identified may have been algorithm results from non-function parentheses, such as from mathematical operations or from script comments. Despite the number of non-package functions, they only represented 11.84% of function calls in the dataset, owing to their rarity: 1 116 596 (91.15%) of these non-package functions were used five or fewer times across the entire dataset.

I then filtered functions via a sampling threshold (electronic supplementary material, figure S1). I retained only functions that were used in at least 0.1% of repos in at least one given calendar month across the study period. This month-based threshold allowed for functions that were newly or historically popular to be included even if they fell below a sampling threshold averaged across the study period. A total of 1194 functions exceeded the sampling threshold.

Finally, I removed 225 820 repos that only contained a single function. These were the equivalent of texts with a single word and contained insufficient data to estimate function diversity.

After processing, the dataset consisted of 3 271 109 scripts from 393 142 repositories created by 223 491 GitHub users. These repos contained a total of 143 409 288 uses of 1194 functions from 135 packages.

#### 2.1.3. Package categories

With the processed dataset, I divided packages into three categories: ‘base' packages, ‘tidyverse' packages and all ‘others'. These categories are briefly described here, but a full listing of function use count, associated package and package category is available as electronic supplementary material, table S1.

The base category included the 14 packages listed as ‘base' in package documentation. Most of these packages are loaded automatically with R and contain code for basic functionality. Packages that were included with R installation but not listed as base (e.g. MASS) were not included in this category. The GitHub dataset included 11 base packages with a total of 562 functions. Base packages and function count (in parentheses) were: ‘base' (353 functions), ‘compiler' (1), ‘graphics' (26), ‘grDevices' (17), ‘grid' (9), ‘methods' (13), ‘parallel' (7), ‘splines' (1), ‘stats' (103), ‘tools' (2) and ‘utils' (30). Three base packages (datasets, stats4 and tcltk) had no used functions in the sub-sampled GitHub data.

The tidyverse is an interrelated collection of community-written R packages that all share a consistent design philosophy [25], including both a core set and a vast array of extension packages. In my processed data, the tidyverse category included 261 functions from 22 packages. This included all eight core tiydverse packages (and function counts): ‘dplyr' (74), ‘forcats' (2), ‘ggplot2' (94), ‘purr' (9), ‘readr' (10), ‘stringr' (19), ‘tibble' (6) and ‘tidyr' (9). In addition, this category included 14 packages associated with the tidyverse: ‘broom' (2), ‘cowplot' (2), ‘ggpubr' (1), ‘ggrepel' (2), ‘glue' (1), ‘haven' (1), ‘httr' (4), ‘jsonlite' (3), ‘lubridate' (8), ‘magrittr' (4), ‘readxl' (2), ‘recipes' (1), ‘rstatix' (2) and ‘rvest' (5).

All non-base and non-tidyverse packages were included in the ‘other' category. This category comprised 371 functions from 102 packages, with half of packages (51) represented in the dataset by a single function. These packages were mostly designed to improve or fill gaps and limitations in R functionality. The ‘shiny' package was the largest package in this category, represented by 60 functions; this package allows the development of interactive web apps within R. Other examples included the ‘data.table' package (21 functions), which increases performance with extremely large databases, the ‘raster' (14) and ‘sf' (8) packages which handle spatial data and ‘igraph' (6), which implements methods and analyses for networks.

### 2.2. Function diversity trends over time

To capture trends in the diversity of function use across the study period, I counted functions used across all repos published in each calendar month (global diversity), as well as a sum of unique functions used within each repository. Additional models and results using diversity estimates that weighted functions based on abundance are provided in the electronic supplementary material.

I modelled trends over time in global and within-repository diversity with generalized additive models (GAMs) using the gam function of the mgcv package [26]. Additive models allow for the fitting of flexible spline terms that can track nonlinear trends through time. All models included one main fixed effect: number of months since January 2014, fit as a thin plate spline. As response variables were counts, these models were fit using a log link function and a negative binomial error distribution. In both models, diversity estimates may have been linked to the number of samples (a sampling effect). To statistically correct for this, I included a sampling covariate in each model. For the global diversity model, this was the number of repositories created in each month; for the within-repository diversity model, this was the number of R scripts in each repository. Both covariates were log-transformed. Model predictions were made using mean covariate values: 3617 repositories for the global model, and 98 scripts for the within-repository model.

I also calculated monthly function diversity estimates separately for ‘base', ‘tidyverse' and ‘other' categories, globally and within-repositories. The global trend over time for each package category was modelled with the structure described above, adding separate splines and intercepts to each of the three categories. As it was possible for a single repository to contain zero functions of a particular category, the within-repository model estimated the probability of a repository including at least one function from a given category (binomial GAM with logit link function). In both cases, the sampling covariates from above were included, with model predictions made at the same values.

### 2.3. Function use trends over time

I established trends in function use over time by using the presence or absence of each function in repos as a response variable in a binomial generalized linear model. Each calendar month was treated as a replicate, with the count of R repos from each month constituting a set of weighted trials. Repos containing a particular function were considered successes and vice-versa. This was conducted separately for each function and modelled as a function of time. I extracted the intercept (reflecting probability of function use in December 2021) and slope (reflecting change in probability over time on a logit scale) from each model. I then repeated these models, but aggregated functions into packages, treating the use of any package-associated function as a success, with separate models for each package. I plotted intercepts and slopes of function and package change visualize patterns, reflecting word shift graphs used in linguistic research [27], except with fully quantitative use probability (or popularity) on the *y*-axis instead of rank order.

Frequency of use is positively linked to stability of use in natural languages [28]. I tested for this pattern in R by modelling the frequency of function use (model intercepts from above) against the magnitude of change in use (absolute value of model slopes from above), interacting with function category (base, tidyverse and other). This was modelled as a linear regression, using intercepts on the logit-link scale as the response variable. This model provided both a statistical test for differing mean use probabilities between function categories, as well as estimating the strength and direction of commonness-change correlations. Statistical differences were estimated *post hoc* using the emmeans and emtrends functions in the emeans package [29].

Additional multi-dimensional methods to visualize and estimate change in function use over the study period are available in the electronic supplementary material.

### 2.4. Change in use within synonymic function groups

As examples of function change over time, I selected three groups of functions that have the same use case (synonymic). These included 12 functions that import tabular-style data (row and column data commonly entered and manipulated in spreadsheet-style software), seven functions that use index matching to combine tabular data objects together and nine functions that reshape existing data objects. These use cases are extremely common in data science. I modelled trends in use over time for each function using a binomial GAM, estimated as the probability that a function would be used in a repository created in a given month. I captured the overall proportional change in function use over the study period by dividing the mean use probability across 2021 by the respective mean probability across 2014.

## 3. Results

### 3.1. Trends in R function diversity

**Figure 1.**
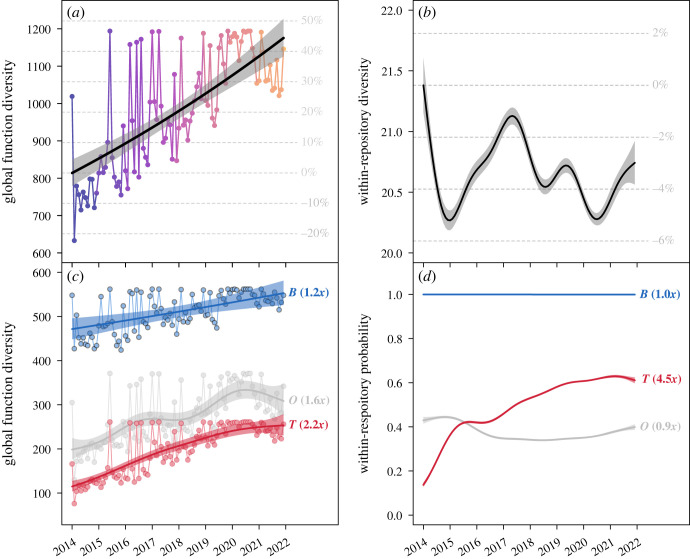
Diversity of R functions in use over time, highlighting (*a*) the average diversity of functions used across all GitHub repositories and (*b*) average function diversity within a repository. (*c*) Trends through time with functions split into three categories: functions from core R installation (base: ‘B’), those from a collection of interrelated community packages (tidyverse: ‘T') and those from all other community packages (other: ‘O'). (*d*) Within-repository trends over time, estimating the probability that a repository used at least one function from a given category. Coloured lines and polygons are mean and 95% confidence intervals, respectively. Grey grid lines in (*a*,*b*) reflect proportional increases or decreases over time relative to January 2014 estimates. Bracketed numbers following each trend in (*c*,*d*) reflect the proportional change for each function category between 2014 and 2021 (e.g. 2.0× = double).

The global diversity of R functions used across all repositories increased linearly over time, ending at *ca* 45% more diverse in December 2021 than January 2014 (figure 1*a*). By contrast, the average diversity of functions used within repositories showed significant but near-zero change (figure 1*b*). Across the study period, the average number of functions used in a repository decreased by 1% to 5% in a cyclical pattern, ending at 3% below 2014 levels.

These diversity trends were not consistent between function categories. The diversity of base R functions over time was largely stable within repositories but increased slightly globally (figure 1*c,d*). A greater diversity of functions from other packages were used across all repositories as time progressed (figure 1*c*), but the probability of use within repositories remained constant at *ca* 0.4 (figure 1*d*). The most profound changes were in the tidyverse category: the overall diversity of tidyverse functions used increased by 2.2 times on 2014 levels (figure 1*c*). There was also an over fourfold increase in the probability that tidyverse functions would be used within a repository (figure 1*d*).

Similar results were found using diversity estimates weighted by function commonness, with two exceptions (electronic supplementary material, figure S2). First, within-repository function diversity reduced by *ca* 7% over time (electronic supplementary material, figure S2B). Second, tidyverse functions showed reductions in global diversity (0.7× on 2014 estimates) and only a 1.2× increase in within-repository diversity (electronic supplementary material, figure S2C-D).

### 3.2. Trends in function and package use over time

**Figure 2.**
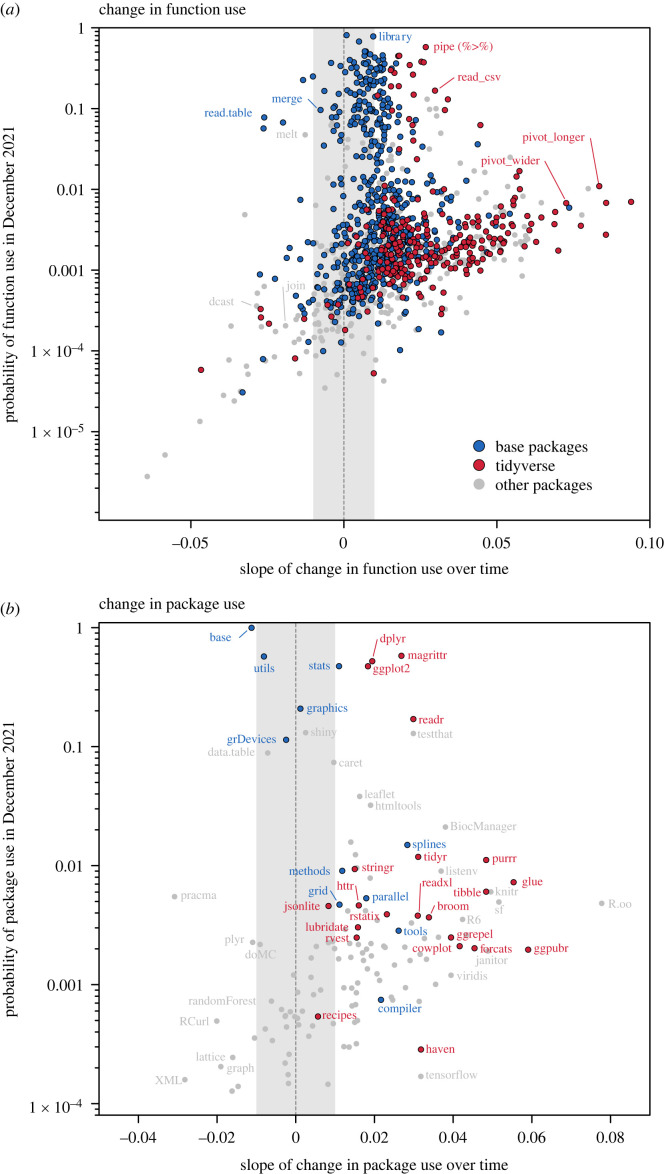
Trends over time in the use of individual R (*a*) functions and (*b*) packages. Plots are ‘word shift graphs', with *y*-axis scores reflecting probability a function was used in a GitHub repository in December 2021, and the slope of change in this probability over time on the *x*-axis. Point colours reflect function category: functions from the base R installation (blue), the tidyverse collection of packages (red) and all other community packages (grey). Some functions from figure 3 are labelled here. Grey dashed line represents a zero slope where function use did not change over time. Shaded grey area highlights an area with small changes that were close to zero. Data and values for each function and package are available in electronic supplementary material, table S1–S2.

Patterns of function commonness followed a roughly lognormal distribution, with an over-abundance of rarely used functions (electronic supplementary material, figure S3), fitting with Zipf's Law of expected word use frequency [30]. On average, base functions were significantly more common than both tidyverse functions (0.787 ± 0.170, *t* ratio = 4.627, *p* < 0.001) and other functions (1.527 ± 0.139, *t* ratio = 10.979, *p* < 0.001), with tidyverse more common than other functions (0.741 ± 0.172, *t* ratio = 4.300, *p* < 0.001). Ninety-eight functions (8.20%) were predicted to occur in greater than or equal to 10% of repositories in December 2021 (figure 2*a*), 82 of which were base functions. Fifteen were tidyverse functions and only one was from an ‘other' package (figure 2*a*). Results for all functions can be found in electronic supplementary material, table S1.

Function composition was strongly structured by time; 34.81% of variation in overall function composition could be explained by time (electronic supplementary material, figure S4). This time-based structuring was true for all package categories when modelled separately: 57.13% of variation in base function composition, 95.02% of tidyverse function composition and 50.63% of other function composition was explainable by time (electronic supplementary material, figure S5).

Just over three-quarters of functions (925: 77.47%) significantly increased in the probability of use over time: 102 decreased (8.54%) and 167 (13.99%) showed no significant trend. Many trends were significant, given large sample sizes, but weak. Using a slope threshold of ±0.01 to reflect a ‘large' change in use, 720 functions increased more than the threshold (60.30%), 55 decreased (4.61%) and 419 had changes below the threshold (35.09%).

While over three-quarters of base R functions showed a significant increase or decrease in use over time (78.11%), only half (46.80%) were large changes: in base packages, large changes comprised seven increases and one decrease (figure 2*b*). Almost all tidyverse functions and packages increased over time; only 19 (7.28%) tidyverse functions, and no packages, had slopes less than zero (figure 2*b*). All tidyverse packages had a positive slope and some tidyverse packages (e.g. dplyr, magrittr, ggplot2 and readr) were as commonly used as the most common base R packages by the end of 2021 (figure 2*b*). Other community packages tended to be more rarely used and had a wider distribution of changes over time, with use probabilities below 0.15 for all packages and functions (figure 2). Most functions from other community packages increased in use over time: 244 (65.77%) significantly and 211 (56.90%) with absolute slopes exceeding 0.01. All bar one of the packages that decreased strongly in occurrence probability were from this other category (figure 2*b*).

Base R functions exhibited an inverse relationship between commonness and change over time (−19.977 ± 9.018, *t* = −2.215, *p* = 0.027: electronic supplementary material, figure S6). This represents a positive commonness-stability relationship. Both tidyverse and other function categories exhibited the opposite pattern, with more common functions exhibiting greater rates of change (electronic supplementary material, figure S6).

### 3.3. Use trends within synonymic function groups

**Figure 3.**
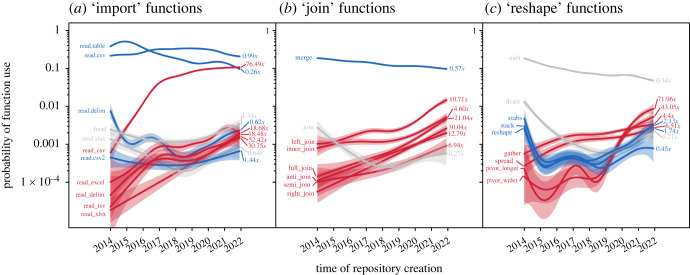
Patterns of use over time within groups of synonymic functions: those that (*a*) import tabular data into R, (*b*) join tabular objects based on index matching and those that (*c*) reshape data objects. *Y*-axis values are the probability that a GitHub repository used a specific function. Functions are coloured based on their category (base, blue; tidyverse, red; other, grey). Right-hand numbers are proportional changes from 2014 to 2021 yearly means (i.e. 2.0× = doubling in probability of use).

In functions that import data, there was a shift towards R users preferring a format of input data rather than a particular function: the strongest trends were in functions that import comma-separated value files (.csv) or file formats from Excel (figure 3*a*). The base function ‘read.csv' maintained its probability of use across the study period (figure 3*a*). The base function that joined data objects together (merge), while still popular by the end of 2021, was slowly being replaced by a collection of six functions from the tidyverse (figure 3*b*). The most used reshape function (melt) is not included in base R (figure 3*c*). Base R functions with similar functionality were rarely used across the study period (figure 3*c*). Functions from the tidyverse were rarely used in 2014 but increased substantially in almost all cases (figure 3). For nine out of 13 tidyverse functions across all three groups, probability of use increased more than tenfold in 8 years, with ‘read_csv' increasing in use over 76 times (figure 3*a*). Notably, no tidyverse functions decreased in the probability of use (figure 3). By contrast, the largest increase in base functions and functions from other packages were ‘stack' and ‘fread', with twofold and one-third increases, respectively (figure 3).

## 4. Discussion

Over an 8-year period, the R programming language has undergone rapid expansion and directional change in function use, driven by the uptake and use of community-created extensions. These patterns of language change are evidence that despite their designed nature, programming languages can change and evolve over time. Overall, I found that change in R has been substantial; some functions, especially those included in the popular tidyverse package collection, experienced more than tenfold increase in use in less than a decade, turning from rarities into core programming verbs. The magnitude of language change in R mirrors observed rapid ‘language-splitting' events [31], raising concerns about a divergence into distinct R dialects that may impact the readability of historical R programs. Such profound change may have flow-on consequences for the continuity of analytic and scientific inquiries codified in R and other programming languages with similar features.

Even though programming languages are designed to interface with computer hardware, and their status as true ‘languages' is debated [32–34], they are designed and used by people. Given a desire for simple execution and comprehension, there is a drive to improve programming languages to make them more intuitive and align with the way humans solve problems [35]. This occurs at the inter-language scale, where programming languages such as Julia were designed to fulfil specific limitations within scientific computing [36]. My results suggest this improvement can occur within languages too. The second most used R function by the end of 2021 was ‘library', which loads an external package of functions, highlighting the strong preference of R users to access and use non-core functionality. Most community packages in my analyses were rarely used across the entire study period, but in 8 years tidyverse functions and packages rose from virtual obscurity to, in some cases, co-dominance. Rather than being rare outliers in a distribution of changing function usage, these patterns are markers of a broader paradigm shift in the building blocks of language that R programmers are using.

R is a ‘new' language, at least relative to the age of natural languages, and rapid diversification and change is expected in new languages from creation and consolidation of new words [13]. We observed patterns of diversification in global function diversity, as well as in individual functions. It appears the R language is still diversifying and increasing in size, potentially driven by cultural selection, a lag between initial function gains and subsequent losses (termed an extinction debt), or by features of R that make the language more susceptible to stochastic drift. Alternatively, there is evidence that rapid change drives fundamental language divisions [31]; the diversification and change in R function use may have arisen from an increasingly large divide between base and tidyverse R ‘dialects'.

### 4.1. Cultural selection

Big data operations with millions or even billions of observations are becoming more common, as are complex analytic processes such as quantitative genetic pipelines, machine learning, cloud computing and Bayesian statistical models. The size and complexity of tasks being performed in R have likely increased commensurately with increases in data size and availability, acting as selective drivers for the development of new functions to accomplish new goals. The lability in function use patterns from other R packages may be an indication of this, as these packages comprise groups of specialised functions (e.g. BiocManager for bioinformatics). Over time, older functions and packages are likely to be superseded by those implementing new analytic techniques or superior implementation of existing techniques (e.g. randomForest versus tensorflow).

The increasing complexity of data analyses may be incentivising users of other statistical tools, such as spreadsheeting software, to learn R, increasing the population size of R speakers. The tidyverse is designed to mirror human sentence structure more closely than the mathematical logic of base R, making it a simple entrance point for new R users [25]. The rapid increase in usage of the ‘read_csv' function may be evidence of large numbers of new R users (figure 3*a*). This function is the default used by the popular RStudio graphical interface for R, which may be a proxy indicator for new R users.

Increasing numbers of new R users, driven by the need for more flexible and powerful statistical tools, may be driving broader, culturally driven change in R away from base functionality and towards the tidyverse. When words under cultural selection increase in popularity, associated words not under direct selection can increase as well: this is called ‘advective change' [7]. There is evidence for advective change in R use, as functions included in the tidyverse increased in aggregate, especially compared with non-tidyverse functions that are nearly identical. As an example, the ‘plyr' and ‘dplyr' packages offer similar functionality and share both a design approach and developers [37,38]. The primary difference is dplyr's inclusion and integration into the tidyverse. Over time dplyr's use increased, while plyr's use dropped (figure 2*b*). These trends need to be interpreted with care. Some function names are shared between the two packages and were assigned to dplyr in my analyses, and observational data can only identify patterns, not processes. Despite this, non-overlapping functions from the two packages mirrored the same trends; plyr functions decreased in use while dplyr functions increased (in all bar one case: electronic supplementary material, figure S7).

### 4.2. Extinction debts

The large increases in overall R function diversity may reflect extinction debts observed in ecological systems, where the actual species extinction is delayed well beyond the causal change in conditions [39]. This pattern has been observed as a lag between expansion and subsequent contraction of natural languages [13], particularly those requiring specialized terms like scientific English [6]. Almost no R functions that passed my sampling filter went completely extinct across the study period, although some (such as ‘read.table' and ‘merge' in figure 3*a,b*) reduced substantially in use. Word use change is driven by positive frequency-dependence, where more popular words become increasingly preferred, creating a feedback loop [12]. As opposed to negative frequency-dependence, which is theorized to stabilize ecological systems and limit species loss [40], these positive feedback loops may cause R to contract in diversity over time, driving lesser-used functions to extinction and continuing language change into the future.

To maintain backwards compatibility of software, programming languages need to be able to understand and process all functions, even those that are not being used anymore. Over time this may lead to bloated installation and documentation size. There were 1997 base R functions used in GitHub repositories that were excluded by my sampling filter: 3.57 times more than were included. Many more functions were used rarely than were used commonly, visible in the distribution of function commonness (electronic supplementary material, figure S3). While language bloat is not currently a problem, it may escalate in the future as new functionality is introduced.

Backwards compatibility within base R is also unlikely to be an issue, but this is not the case in community-written packages. Of the 19 318 packages listed on R's official package repository, at time of writing, 5567 (28.81%) had not been updated since R v.4.0 released in April 2020. Some of these may be orphaned, with no active developers, and may have incompatibilities with subsequent changes to R or with other interdependent packages that have been updated more recently. R programs written using these abandoned packages may be non-functional, or even incomprehensible to current R users.

### 4.3. Stochastic drift

Population sizes for programming languages are small; they are learned only for specialized purposes, and direct communication is between a user and a computer. This may make them more susceptible to random drift, like genetic evolution patterns in small populations [41]. R's design use, scientific and statistical computing, may be especially vulnerable to communicative rifts between users, as programs are often written by individuals or small groups of users in corporate or research silos, limiting the cross-population flow of cultural forces. The generation time of R users is also likely much shorter than natural languages, which is tied to lifespan. Instead, turnover is linked to time spent in related industries, which may be as long as an entire working life, or as little as the length of a degree or PhD.

While a large data source, GitHub repositories likely represent a biased subset of R users. Many R programs are never made publicly accessible, and GitHub users may reflect a ‘power user' subpopulation more willing to adapt and change their use patterns, as well as develop new R packages. This subpopulation may be more susceptible to cultural forces that drive trends in function use. Many of the GitHub users in the dataset only published a single repository, likely increasing the influence of inter-user variance on observed diversification. There are, however, similarities between published R repositories and published authorial works in natural languages: corpora of published books underpin many studies of language evolution (e.g. [42]).

### 4.4. Popularity of the tidyverse

The most substantial changes in R use were in the tidyverse, which brings a unique dialect of functionalization to R; tidyverse packages provide a number of discrete functions that replace a single base function. The synonymic ‘join' functions outlined in figure 3*b* showcase this. Base R's ‘merge' function offers the functionality of the six tidyverse functions via internal arguments. Base R functions tend to be flexible tools that can be modified to achieve specific outcomes, while the tidyverse provides sets of specific functions that each accomplish one goal. As a coarse metaphor in natural language terms, base R tends to provide general verbs, such as ‘run', and allows them to be modified by adjectives (e.g. ‘fast', ‘slow’ and ‘relaxed'). The tidyverse instead tends to provide a larger series of more specialized verbs (i.e. ‘sprint', ‘jog’ and ‘amble').

This functionalization trend explains much of the observed increase in diversity of R functions over time. Almost all increases in the diversity of R functions used came from non-base groups, and the tidyverse exceeded the growth in all other community packages combined. Not only were repositories over four times more likely to use tidyverse functions by the end of 2021, but GitHub repositories that used the tidyverse, on average, used *ca* 15% more functions than repositories with no tidyverse use (electronic supplementary material, figure S8). When I weighted diversity estimates towards more common functions, the rapid increase in tidyverse diversity over time virtually disappeared (electronic supplementary material, figure S2). In aggregate, these results suggest change in R use over time is being driven by R users drawing from a larger spectrum of more specialized functional tools.

Rapid language change is linked to splits in languages [31], but my results provide inconclusive evidence that R is dividing into base and tidyverse ‘dialects'. Function change over time was detectable across all package categories, including base R packages (electronic supplementary material, figure S5), with the majority of base R functions also increasing in commonness (figure 2*a*). This suggests that despite trends towards the tidyverse, R users who rely on base functions are still common in the population, even in 2021. While this could suggest separate dialects are forming, base R functions showed the expected pattern between commonness of use and stability of use, where more common functions were also more stable. This suggests the core functionality provided by base R is still relied upon by most users. However, tidyverse and other categories showed opposite commonness-stability trends; more commonly used functions in these groups were more labile, with greater proportional increases or decreases (electronic supplementary material, figure S6). There appears to be strong and systematic selection of some tidyverse functions over base functions among R users, but further research into potential dialectic divide is needed.

### 4.5. Functions as language evolution indicators

My analysis focused specifically on functions and excluded all other types of R objects, which may have amplified the signal of language change over time. Notably, I excluded the names of non-function objects, which are reflective of nouns in natural languages. Focussing solely on verbs may have amplified the signal of language evolution, as noun conventions could both increase variation (e.g. user variation in object naming conventions) as well as decreasing change over time (e.g. consistent use of common object names like ‘data', ‘a', ‘x').

There may also be features of R that are more resistant to change than functions, such as sentence structure in natural languages [43,44], or less resistant, such as grammatical features [45]. One such grammatical shift in R is the transition towards ‘piping', linking R functions together via programmatic semicolons. This fundamentally shifts the grammar of R as a language, even if it does not alter functions directly. Pipes were initially part of the tidyverse (and marked as such in my analysis) but were included in base R from 2020. By the end of 2021, pipes were observed in roughly half of R repositories, *ca* 12-fold increase on 2014 usage that made pipes the fourth most common function (figure 2*b*). Despite this, I targeted an aspect of R grammar to identify functions during data collection: that function calls are always paired with parentheses. This grammar has not changed over time, and there currently no competing methodology for this approach. My results suggest that non-functional features of programming languages do not appear to be innately resistant to change, so long as alternative structures are feasible and available to be culturally selected by users.

## 5. Conclusion

Programming languages are a way to express a mental plan for a computer to implement. While programmers do not need to design language around the imperfect interpretation of a human reader, they are still humans writing language. The rapid transition in R towards the tidyverse suggests that while R's core design may have been internally consistent, it is being selected against by the population of users, accommodated by the easy publication and incorporation of community-written packages. R's design has facilitated rather than restricted this change, as its stated role is an ‘environment’ for developing and implementing new data analysis methods, rather than a rigid set of programming tools [46]. This rapid change in R raises some concerns about the continuity and readability of programs written over time. Even so, allowing programming languages to evolve naturally may be a way to crowd-source language improvements, leveraging the natural tendency of human speakers to converge and refine languages over time [6,8]. My results suggest that both programming language design and study should consider linguistic understanding of human language preferences and the processes that drive language evolution.

## Data Availability

R code to access GitHub repositories, process function data and reproduce all analyses, figures, and tables is published online at https://doi.org/10.5281/zenodo.7758108 [47]. Given the complexity of data access, the processed dataset is provided in full at https://doi.org/10.5061/dryad.h18931zrg [48]. These data are summarized in electronic supplementary material, tables S1–S4 and accompanying metadata [49].
